# SL-Cloud: A Cloud-based resource to support synthetic lethal interaction discovery

**DOI:** 10.12688/f1000research.110903.2

**Published:** 2022-12-22

**Authors:** Bahar Tercan, Guangrong Qin, Taek-Kyun Kim, Boris Aguilar, John Phan, William Longabaugh, David Pot, Christopher J. Kemp, Nyasha Chambwe, Ilya Shmulevich

**Affiliations:** 1 Institute for Systems Biology, Seattle, WA, 98109, USA; 2 General Dynamics Information Technology, Rockville, MD, 20852, USA; 3 Division of Human Biology, Fred Hutchinson Cancer Research Center, Seattle, WA, 98109, USA; 4 Institute of Molecular Medicine, Feinstein Institutes for Medical Research, Manhasset, NY, 11030, USA

**Keywords:** synthetic lethality, cloud computing, cancer genomics, cancer dependency, systems biology, functional genomics

## Abstract

Synthetic lethal interactions (SLIs), genetic interactions in which the simultaneous inactivation of two genes leads to a lethal phenotype, are promising targets for therapeutic intervention in cancer, as exemplified by the recent success of PARP inhibitors in treating BRCA1/2-deficient tumors. We present SL-Cloud, a new component of the Institute for Systems Biology Cancer Gateway in the Cloud (ISB-CGC), that provides an integrated framework of cloud-hosted data resources and curated workflows to enable facile prediction of SLIs. This resource addresses two main challenges related to SLI inference: the need to wrangle and preprocess large multi-omic datasets and the availability of multiple comparable prediction approaches. SL-Cloud enables customizable computational inference of SLIs and testing of prediction approaches across multiple datasets. We anticipate that cancer researchers will find utility in this tool for discovery of SLIs to support further investigation into potential drug targets for anticancer therapies.

## Introduction

The concept of synthetic lethality (SL) refers to interactions between two genes in which loss of function of either gene alone does not impair cell viability, whereas inhibition of both genes is lethal ( O’Neil *et al.*, 2017). In the context of anticancer therapy, two genes are synthetic lethal if mutation of either alone is compatible with viability but mutation of both leads to death ( Kaelin, 2005). It can be extended to the broader concept that the alteration of one gene is compatible with viability, but the alteration of both leads to death or lower viability. These interactions are attractive for designing cancer therapies, as targeting a gene whose synthetic lethal partner is permanently inactivated in cancer cells but exhibits wild-type expression in healthy cells should selectively kill cancer cells. The synthetic lethal interaction (SLI) between the poly (ADP-ribose) polymerase ( *PARP*) genes and BRCA deficiency (functional loss of either B *RCA1* or *BRCA2*) is the first successful clinical application of the SL concept ( Fong *et al.*, 2009; Lord & Ashworth, 2017). Subsequent functional screens have proposed other synthetic lethal pairs, including the SWI/SNF chromatin remodeling complex members *SMARCA2*- *SMARCA4* ( Hoffman *et al.*, 2014) and *ARID1A-ARID1B* ( Helming *et al.*, 2014), as well as the Werner syndrome RecQ-like helicase ( *WRN*) gene in *MYC* overexpressing cancers ( Moser *et al.*, 2012) and microsatellite unstable cancers ( Chan *et al.*, 2019; Kategaya *et al.*, 2019; Lieb *et al.*, 2019). Although SL-based therapeutics are promising, other drugs for clinical use designed using an SL-based rationale are still under development. There is, therefore, a continued need to discover additional synthetic lethal gene pairs and to develop automated methods that use various data types to predict clinically relevant synthetic lethal pairs that can be nominated for further testing and therapeutic development ( Huang *et al.*, 2020).

Functional screening using siRNA/shRNA technology or, more recently, CRISPR-based targeting libraries, is a leading method of SLI discovery ( O’Neil *et al.*, 2017). However, identifying robust synthetic lethal gene pairs is challenging, in part due to biological factors such as genetic and epigenetic heterogeneity and incomplete penetrance, i.e. context-dependent SL ( Chan *et al.*, 2010; Henkel *et al.*, 2019; Nijman & Friend, 2013; Ryan *et al.*, 2018). To complement functional screening efforts, multiple computational prediction strategies have been pursued (reviewed by ( O’Neil *et al.*, 2017). Early approaches inferred SLIs in humans via ortholog mapping based on genetic interaction networks from experimentally-tractable model organisms such as *Saccharomyces cerevisiae* ( Conde-Pueyo *et al.*, 2009; Kirzinger *et al.*, 2019; Srivas *et al.*, 2016) and *Mus musculus* ( Gurley & Kemp, 2001). Alternative strategies rely on the integrated analysis of multi-omics profiling and functional screening of patient-derived or cancer cell line-based datasets to predict SLIs. These approaches use statistical and/or heuristic methods, such as implicating SL gene pairs via mutually exclusive loss-of-function mutations, shared pathways or protein complex membership ( Das *et al.*, 2019; Jerby-Arnon *et al.*, 2014; Lee *et al.*, 2018; Liany *et al.*, 2020; Wappett *et al.*, 2016; Ye *et al.*, 2016). Furthermore, to facilitate interactive exploration of predicted SLIs, several web portals or SLI databases have been published, such as Syn-Lethality ( Li *et al.*, 2014), SynLethDB ( Guo *et al.*, 2016), the Synthetic Lethality BioDiscovery Portal (SL-BiodP) ( Deng *et al.*, 2019), the Cancer Genetic Interaction Database (CGIdb) ( Han *et al.*, 2019), and, more recently, SynLeGG ( Wappett *et al.*, 2021). These tools present pre-computed synthetic lethal pairs based on the most comprehensive datasets available at the time of publication. This necessarily excludes potential SLIs discoverable by either algorithmic advances or developments in functional screening technologies in terms of scope and throughput. Additionally, there is limited flexibility to explore the existing set of putative SLIs or to change any parameters in the prediction algorithms to better understand how the SL inference was made. 

There is significant complexity in these prediction approaches because of the need to manage the amount of data on which predictions are based, and the need to select the most appropriate datasets and tools without objective criteria to determine how well any given approach performs. Here we provide a cloud-based framework, Synthetic Lethality Cloud (SL-Cloud), that enables the inference of SL gene pairs from multiple prediction approaches simultaneously on the same datasets. As compared to other current computational approaches for SLI prediction, we provide customized scripts and a facile connection to large public data resources, simplifying the use of publicly available data that can be repurposed for SLI prediction. SL-Cloud is a new component of the Institute for Systems Biology Cancer Gateway in the Cloud ( ISB-CGC) resource, a data science infrastructure that provides secure access to a large, comprehensive, and expanding collection of cancer research data ( Reynolds *et al.*, 2017). The software draws on and adds to the ISB-CGC resources, enabling the identification of potential SL gene pairs for a specific cancer of interest. We present here an overview of our implementation of the SL-Cloud resource, as well as use cases that showcase the utility of the resource for SL research.

## Methods

### Implementation

**Figure 1.**
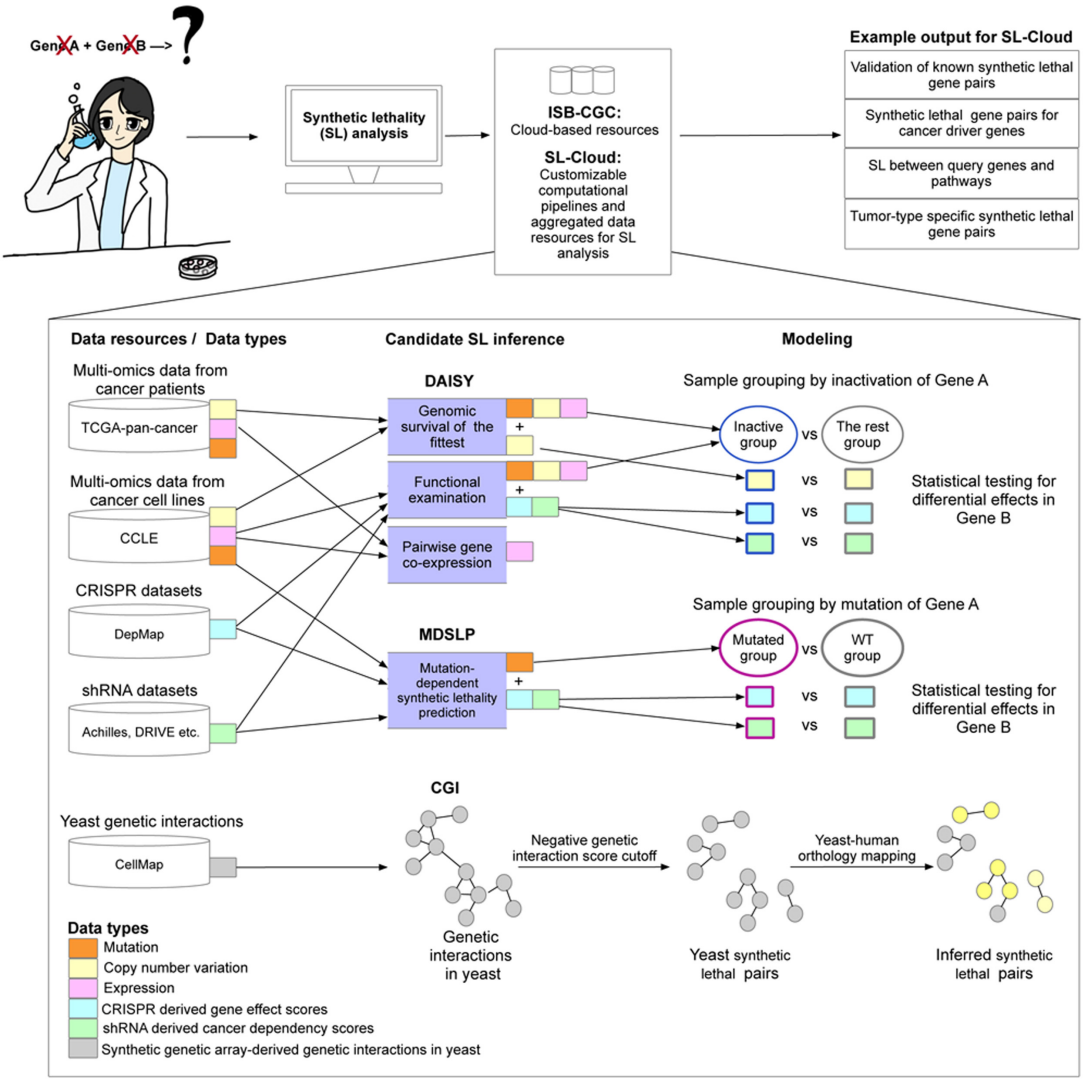
Overview of the Synthetic Lethality Cloud (SL-Cloud) framework. Schematic overview of the computational workflows and data resources aggregated in this framework to facilitate investigation of synthetic lethal interactions. Users with specific research questions (top-left) reuse inference workflows from the provided scientific computing notebooks to query public cancer genomics datasets from the vast resources provided by the ISB-CGC and additional datasets pre-processed in SL-Cloud. Three candidate synthetic lethal pair inference workflows were implemented, including the DAta-minIng SYnthetic lethality identification (DAISY), mutation-dependent synthetic lethality prediction (MDSLP), and conserved genetic interaction (CGI) workflows. Different data resources and data types were used for different inference workflows. For example, the DAISY and MDSLP workflows rely on statistical testing over the multiple omics data and functional screening data such as CRISPR and shRNA datasets for human cancers, whereas the CGI workflow is based on an ortholog mapping of the SL interactions identified in yeast.

SL-Cloud aggregates commonly used public data resources relevant for SL inference and integrates them with analysis workflows to infer SLIs by leveraging the cloud-based resources stored on the ISB-CGC platform ( Figure 1). These three components (summarized below) represent an ecosystem that integrates software and data to enable the large-scale prediction of SLIs from existing cloud-hosted datasets.

### Institute for Systems Biology Cancer Gateway in the Cloud (ISB-CGC).

ISB-CGC, a part of the National Cancer Institute (NCI) Cancer Research Data Commons, hosts derived data in Google Cloud Platform BigQuery tables, providing gene expression, mutation, copy number alteration, methylation, protein levels and other molecular characteristics for a broad range of cancers, such that these datasets can be rapidly analyzed using the power of cloud computing ( Bleich, 2022; Reynolds *et al.*, 2017). Google BigQuery is a columnar data warehousing solution that provides fast access through Structured Query Language (SQL)-based queries. The ISB-CGC resource contains over 1000 distinct derived data tables that are findable through both a BigQuery Table Search interface as well as through the native Google BigQuery interfaces. On this platform, users can analyze multiple large-scale datasets solely or in combination with their own datasets while circumventing the need to download and maintain a local copy of these petascale datasets, or to perform extensive data management tasks. We have designed SL-Cloud as a component of ISB-CGC so that we can leverage ISB-CGC’s broad availability, co-location of robust computational resources, and democratized access to raw and derived cancer research data. SL-Cloud allows cancer researchers access to key datasets and workflows to enable the identification of potential synthetic lethal gene pairs for a specific cancer of interest.

### Large-scale datasets relevant for SL Prediction

A key feature of SL-Cloud is the aggregation of key cancer multi-omics datasets for SL inference in a single framework that facilitates integrative analysis. For SL-Cloud, we created and mined the relevant large-scale, publicly available multi-omics and functional screening datasets on ISB-CGC such as The Cancer Genome Atlas (TCGA) ( Hutter & Zenklusen, 2018) patient-level data on somatic mutations, gene expression, and copy number alterations across 33 cancer types, ( Figure 1) (for details see Table 1, which includes the URLs for specific resources). We identified additional public data resources that were pertinent for SL inference but previously unavailable on ISB-CGC. These datasets include a genetic interaction dataset TheCellMap derived from model organism interaction screens ( Costanzo *et al.*, 2016), and human pan-cancer cell line molecular characterization and functional screening datasets, primarily from the Cancer Cell Line Encyclopedia (CCLE) and the Cancer Dependency Map (DepMap) initiative ( Dempster *et al.*, 2019; Ghandi *et al.*, 2019; McFarland *et al.*, 2018; Meyers *et al.*, 2017). Building on the infrastructure of the ISB-CGC, we established a new SL-focused cloud resource within ISB-CGC that incorporates the most relevant datasets for SLI prediction ( Table 1).

**Table 1.** Publicly available cancer genomic and molecular profiling datasets relevant for SL inference.

Data	Data Resource	Data Type	Data/Table	Google Bigquery Table ID	Approach	Reference	Link (Original Data Resource Address)
TCGA omics data	TCGA (The Cancer Genome Atlas)	Copy number variation	all_data_by_genes_ whitelisted.tsv	isb-cgc-bq.pancancer_atlas. Filtered_all_CNVR_data_by_ gene	DAISY	Hutter & Zenklusen, 2018	https://www.synapse.org/#!Synapse:syn5049514
		Gene expression	EBPlusPlusAdjustPANCAN _IlluminaHiSeq_RNASeqV2. geneExp.tsv	isb-cgc-bq.pancancer_ atlas.Filtered_EBpp_ AdjustPANCAN_IlluminaHiSeq_ RNASeqV2_genExp	DAISY		https://api.gdc.cancer.gov/data/3586c0da-64d0- 4b74-a449-5ff4d9136611
		Mutation	Pancan.merged. v0.2.5.filtered.maf.gz	isb-cgc-bq.pancancer_atlas. Filtered_MC3_MAF_V5_one_ per_tumor_sample	DAISY		https://api.gdc.cancer.gov/data/c946eefc-20a0- 4277-a6da-fe42ed4d793a
CCLE omics data	CCLE (Cancer Cell Line Encyclopedia)	Copy number variation	CCLE_gene_cn.csv	isb-cgc-bq.DEPMAP.CCLE_ gene_cn_DepMapPublic_ current	DAISY	Ghandi *et al.,* 2019	https://ndownloader.figshare.com/files/24613352
		Gene expression	CCLE_expression.csv	isb-cgc-bq.DEPMAP. CCLE_gene_expression_ DepMapPublic_current	DAISY		https://ndownloader.figshare.com/files/24613325
		Mutation	CCLE_mutations.csv	isb-cgc-bq.DEPMAP.CCLE_ mutation_DepMapPublic_ current	DAISY / MDSLP		https://ndownloader.figshare.com/files/24613355
		Sample information	sample_info.csv	isb-cgc-bq.syntheticlethality. sample_info_TCGAlabels_ DepMapPublic_20Q3	DAISY / MDSLP		https://ndownloader.figshare.com/files/24613394
CRISPR based gene effects	DepMap (The Cancer Dependency Map)	Gene effect	Achilles_gene_effect.csv	isb-cgc-bq.DEPMAP.Achilles_ gene_effect_DepMapPublic_ current	DAISY / MDSLP	DepMap, 2020; Dempster *et al.,* 2019; Ghandi *et al.,* 2019; Meyers *et al.,* 2017	https://ndownloader.figshare.com/files/24613292
shRNA based gene effects	Achilles DRIVE	Gene dependency score	D2_combined_gene_dep_ scores.csv	isb-cgc-bq.DEPMAP. Combined_gene_dep_ score_DEMETER2_current	DAISY / MDSLP	McFarland *et al.,* 2018	https://ndownloader.figshare.com/files/13515395
Genetic interactions in yeast	TheCellMap	Genetic interaction scores	Raw genetic interaction datasets: Pair-wise interaction format.zip	isb-cgc-bq.supplementary_ tables.Constanzo_etal_ Science_2016_SGA_Genetic_ Interactions	CGI	Costanzo *et al.* 2016	https://thecellmap.org/costanzo2016/

### Synthetic lethal interaction prediction workflows

We implemented SLI inference workflows and distributed them as a set of Jupyter notebooks that use functions from Python scripts provided with this resource. The notebooks offer code optimization and integration with the ISB-CGC through the BigQuery interface to access the relevant pre-processed large-scale cancer genomics datasets described above through embedded SQL queries. Importantly, the workflows can be tailored to individual cancer researcher needs by copying the existing code and optimizing it to their specific use case. We present here a brief overview of three example workflows implemented (for technical details see accompanying documentation on the project GitHub page, the url is available in the Data and software availability section).

**The DAta-minIng SYnthetic lethality identification workflow (DAISY):** This previously published workflow is re-implemented using up-to-date, large-scale data resources as described above ( Figure 1; Table 1) ( Jerby-Arnon *et al.*, 2014). DAISY applies multiple inference procedures that include:

- **genomic survival of the fittest** (SoF): the detection of infrequently co-inactivated gene pairs by using somatic mutation, copy number alteration and gene expression data
- **functional examination** (FunEx): the identification of gene pairs in which inactivation or over-activation of one gene induces essentiality of a partner gene - using functional screening data
- **pairwise gene co-expression**: the detection of significantly positively correlated gene pairs - thereby implicating genes in related biological functions

Inference of synthetic dosage lethality (SDL), whereby overactivation of one gene causes its interaction partner to become essential for cell viability, is also implemented in DAISY. DAISY SL predictions are gene pairs that are found by all three inference modules. Each individual module can also provide evidence of SL potential independently. The workflow, as we have implemented it, enables users to list predicted synthetic lethal pairs from each workflow, for each dataset, and aggregate them or use them independently. The DAISY workflow also enables users to perform pan-cancer or tissue type-specific analyses by tuning to the specific biological question being examined.

**Mutation-dependent synthetic lethality prediction (MDSLP)**: This workflow combines mutation and functional screening data to infer SL pairs from cancer cell line data. The MDSLP workflow is based on the rationale that, for tumors with mutations that have an impact on protein expression or structure (functional mutation), the knockout effects or inhibition of a partner target gene show conditional dependence for the mutated molecular entities ( Figure 1). Leveraging the public cancer cell line datasets including gene mutation data from CCLE, and functional screening data generated by either shRNA or CRISPR technology from DepMap ( Dempster *et al.*, 2019; Ghandi *et al.*, 2019; McFarland *et al.*, 2018; Meyers *et al.*, 2017), we integrated these data modalities to evaluate mutation-based conditional dependence. This workflow enables users to statistically test whether the knockout or knockdown effects for one gene will be altered if another gene is mutated in specific contexts, such as in pan-cancer, or tumor type-specific cell lines. The increase in gene knockout or knockdown sensitivity provides evidence to support potential SLIs.

**Conserved genetic interaction (CGI) workflow:** We implemented this workflow based on published methods described in ( Srivas *et al.*, 2016). The CGI workflow leverages cross-species conservation to infer experimentally derived SLIs in yeast to predict relevant synthetic lethal pairs in humans. For SL-Cloud, we downloaded and preprocessed TheCellMap dataset, the most comprehensive *S. cerevisiae* genetic interaction network inferred from synthetic genetic array (SGA) screens from ( Costanzo *et al.*, 2016) (see details in Table 1). Genetic interactions are inferred if the combined effect of a double mutant on cell viability differs from that of the combination of single mutant effects. SLIs are defined in this context as negative genetic interactions in which the cell viability of a double-mutant yeast colony is lower than that of the respective single-mutant colonies. We provide the inferred SLIs in humans by yeast-to-human ortholog mapping.

### Operation

SL-Cloud workflows are implemented in a set of Python notebooks that can be edited and run via a Jupyter notebook interface. All the requirements are specified in the respective Jupyter notebooks. Users can also access and run the SL-Cloud workflows in mybinder.org, a platform that allows users to run the implementations without installing any libraries or downloading code from a GitHub repository (see setup and operation instructions here).

## Use cases

SL-Cloud facilitates custom analyses demonstrated in workflows for particular research questions or disease contexts. Additionally, this framework provides extensibility by virtue of the modular design of the base framework shown in Figure 1. End-users can combine high-quality public data with their own laboratory-generated data to extend integrated analyses more easily without the need to download and pre-process large-scale public cancer genomics data. In the following sections, we describe specific use cases.

### In silico validation of known or suspected SLIs

**Figure 2.**
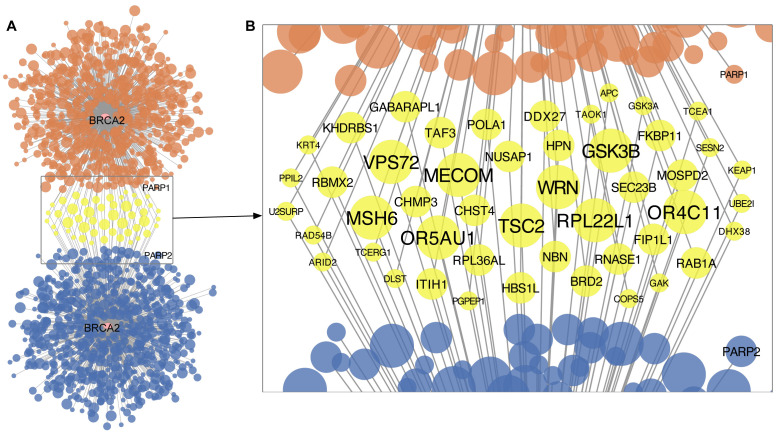
Predicted synthetic lethal interaction partners of *BRCA2* based on cancer cell line dependency datasets. **A.** Network-based representation of predictions generated by the mutation-dependent synthetic lethality prediction (MDSLP) using either shRNA (upper panel, orange) or CRISPR (lower panel, blue) functional screening datasets. **B.** Potential synthetic lethal partners of *BRCA2* as predicted by both the CRISPR and shRNA functional screening data. Each node represents a gene, and edges represent potential synthetic lethal interactions. The node color encodes overlap between the gene-dependency assay type, with yellow representing synthetic lethal pairs predicted in both the CRISPR data and the shRNA functional screening data. The node size and font size indicate the strength of the statistical relationship, with high-confidence pairs having larger node sizes or font sizes. FDR levels: < 0.01 (largest), < 0.05 (median), and <0.1 (smallest).

Synthetic lethality between *BRCA1/2* and *PARP1/2* is well documented and is the rationale behind the design of PARP inhibitors such as olaparib, rucaparib, and niraparib ( Ashworth & Lord, 2018). These agents are approved for treating *BRCA*-mutated ovarian cancer and advanced breast cancer. To perform an *in silico* validation analysis of this well-established SLI, we applied MDSLP to gene mutation and functional screening data from pan-cancer cell lines ( Dempster *et al.*, 2019; Ghandi *et al.*, 2019; McFarland *et al.*, 2018; Meyers *et al.*, 2017). As shown by the MDSLP-shRNA workflow and consistent with our expectations, functional mutations of *BRCA2* showed significant sensitivity to *PARP1* knockdown (two-sided t-test, *P* < 0.01, FDR < 0.1). We applied the same workflow to gene essentiality data derived from CRISPR screens, but did not find this expected interaction. The MDSLP workflow using CRISPR-derived datasets revealed that the functional mutation of *BRCA2* shows a synthetic lethal partnership with *PARP2* ( *P* < 0.01, FDR < 0.05). shRNA-derived and CRISPR-derived *BRCA2* synthetic lethal pairs showed limited overlap ( Figure 2A). Only 6.6% (48 out of 729) of the BRCA2-related synthetic lethal pairs nominated from shRNA-derived inference with a threshold of FDR < 0.1 were also predicted using CRISPR essentiality screens. Of the 1433 potential partner genes predicted by any of the resources, only 48 partner genes were predicted by two resources ( Figure 2B). Of these, *WRN, TSC2, RPL22L1* showed the most significance with both CRISPR-derived and shRNA-derived inference (FDR < 0.01 for both inference procedures).

**Figure 3.**
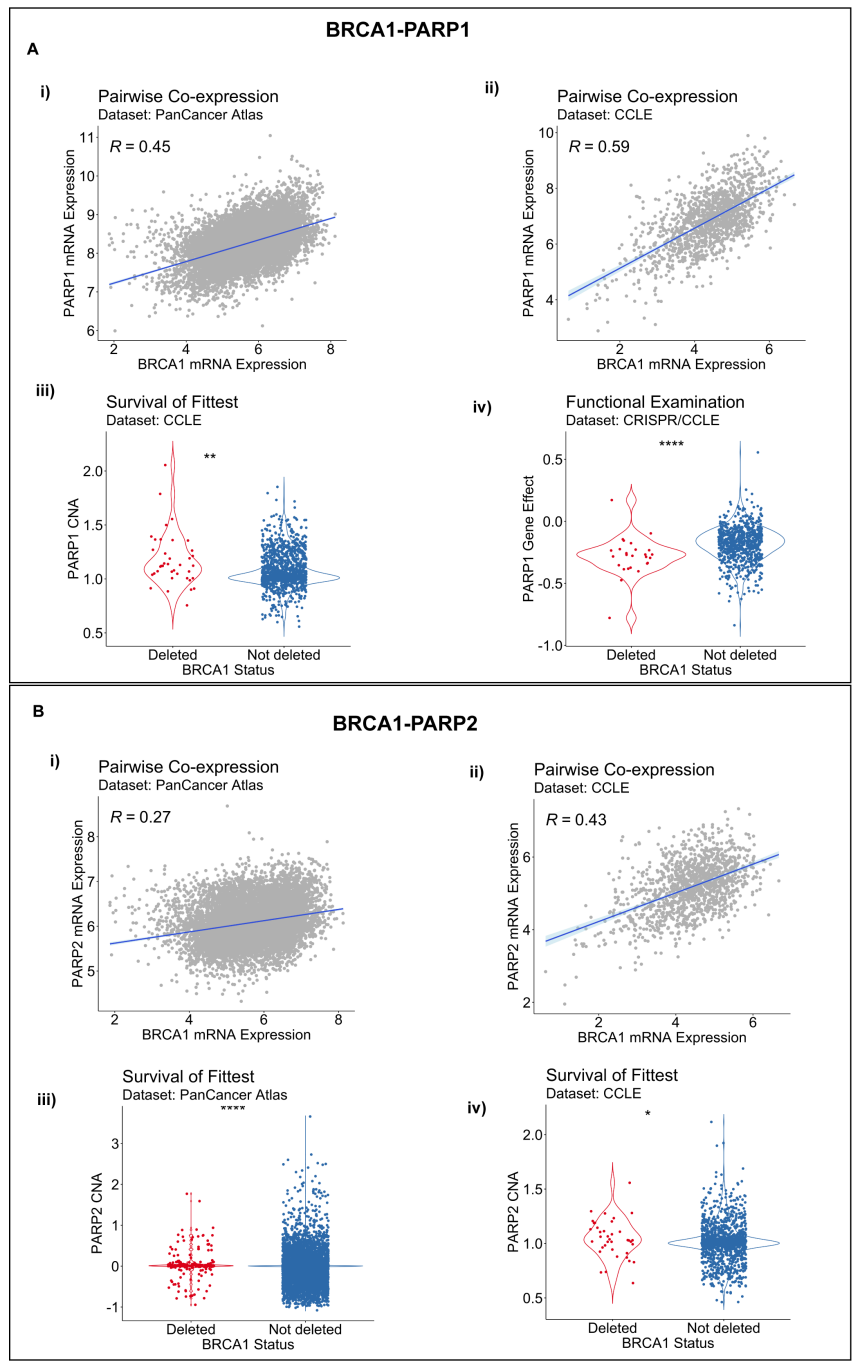
Evidence for BRCA1-PARP1/2 synthetic lethal relationship based on the DAta-minIng SYnthetic lethality identification (DAISY) workflow. **A**. Evidence for a *BRCA1-PARP1* synthetic lethal relationship by pairwise co-expression in i) Pan-Cancer Atlas and ii) CCLE datasets respectively; iii) by survival of the fittest in in CCLE data; and iv) by functional examination in DepMap CRISPR. **B**. Evidence for a *BRCA1-PARP2* SL relationship by pairwise co-expression in i) Pan-Cancer Atlas and ii) CCLE datasets and by survival of the fittest in iii) Pan-Cancer Atlas and iv) CCLE datasets. *R,* Spearman correlation coefficient; p, P value by the one-sided Wilcoxon rank-sum test; * *P* < 0.05; ** *P* <0.01; **** *P* < 0.0001.

Interestingly, we did not predict any *BRCA2-*related synthetic lethal pairs from the other two workflows implemented in SL-Cloud. *BRCA2* has no yeast homolog and, therefore, conserved interactions could not be inferred by the CGI workflow. DAISY nominated no synthetic lethal partners for *BRCA2* with its default settings but predicted potential *BRCA1-PARP1/2* SLIs across all three of its component inference modules with non-default parameter settings. Both gene pairs, *BRCA1-PARP1* and *BRCA1-PARP2*, showed statistically significant co-expression, with their correlation coefficients ranging from 0.26 to 0.59 across patient-derived and cancer cell line datasets [ Figure 3A(i,ii) and 3B(i,ii)] ( *P* < 0.01). In addition, we found statistical support for a *BRCA1* and *PARP1/2* SLIs by the SoF inference procedure [ Figures 3A(iii) and 3B(iii,iv)] ( *P* < 0.05), whereas the FunEx module found statistical support for a *BRCA1-PARP1* SLI [ Figure 3A(iv)] ( *P* < 0.01), but not for a *BRCA1-PARP2* SLI, based on the cancer cell line gene-dependency CRISPR or shRNA datasets. In summary, the *BRCA1-PARP1* interaction was supported by all three DAISY inference modules, whereas only two modules supported the *BRCA1-PARP2* interaction.

This example demonstrates how SL-Cloud facilitates the exploration of the SLIs for a particular gene by using orthogonal SLI prediction workflows and multiple datasets to assess the stability and reproducibility of particular SLIs of interest. For the established SLI between BRCA deficiency and PARP1/2 enzymes, we saw variation in the output of multiple prediction approaches and datasets in confirming this *bona fide* SLI. These analyses highlight some of the challenges related to SL prediction, including unaccounted for variation resulting from differences in the technology and size of the datasets used to make the SL prediction, and the implicit or explicit assumptions made by the underlying analytical approaches. This example shows how SL-Cloud can be applied to enable researchers to explore a particular SLI of interest in different datasets or using different prediction approaches.

### Pathway-based SL analysis

SLI partners tend to form functional interaction networks ( Costanzo *et al.*, 2016; Jerby-Arnon *et al.*, 2014). For example, Ku *et al.* reported that synthetic lethal screen hits are more robust at the pathway rather than at the gene level ( Ku *et al.*, 2020). To demonstrate pathway-based SLI discovery, we analyzed SLI-related genes in the DNA damage and repair (DDR) pathway. DDR deficiency due to loss-of-function alterations by mutation, deletion, or epigenetic silencing is prevalent across lineages affecting approximately 33% of all cancers in TCGA ( Knijnenburg *et al.*, 2018). Impaired DDR leads to genomic instability, and tumors exhibiting DDR loss are prone to DNA-damaging agents and, therefore, potentially vulnerable to inhibitors that target compensatory DDR pathways via a synthetic lethal mechanism ( Lord & Ashworth, 2012). Using a well-curated set of 276 genes annotated for involvement in DNA damage repair from ( Knijnenburg *et al.*, 2018) we predicted synthetic lethal partners from the three workflows described above ( Figure 1).

**Figure 4.**
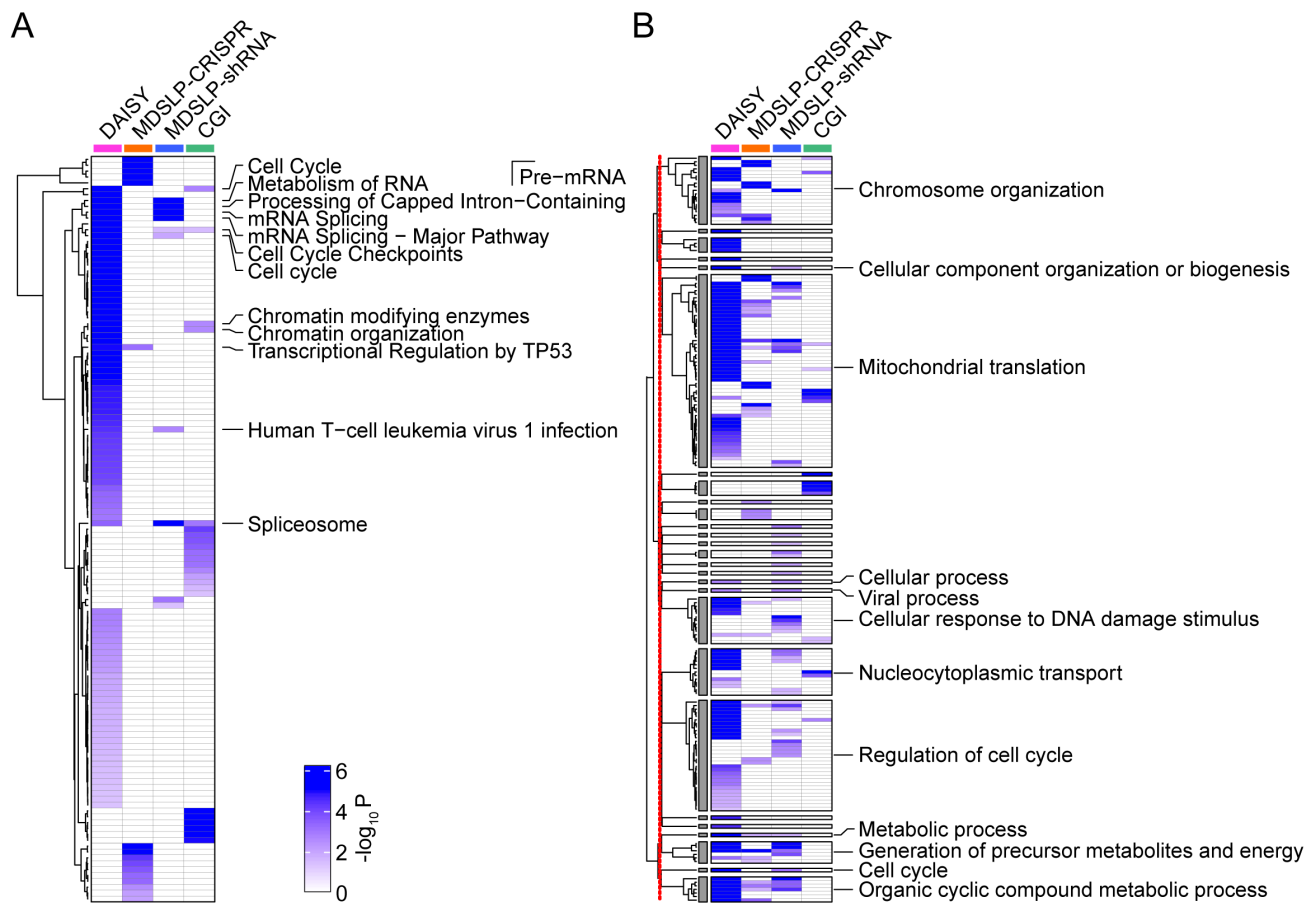
Pathway enrichment of predicted synthetic lethal partners of DNA damage repair (DDR) genes. Heatmaps depicting **A.** the KEGG or REACTOME pathway and **B.** Gene Ontology Biological Process (GOBP) enrichment for predictions made using four different approaches (columns). Increasing color intensity represents increasing statistical significance ( *P* < 0.05, calculated by a hypergeometric test) for enrichment. Pathways or GOBPs were labeled if they were enriched by synthetic lethal partner genes identified by at least two prediction approaches. The redundant GOBPs were further reduced by REVIGO. The 398 GOBPs enriched by at least one approach were reduced to 172 GOBPs based on their semantic similarities, and then summarized into 27 representative groups whose enrichment significance is represented in the heatmap. Clustering analysis was performed for GOBPs inside and outside of the 27 representative groups, separately. The red line down the left side of panel B indicates the separation between the clustering analyses. DAISY, data mining synthetic lethality identification workflow; MDSLP-CRISPR, mutation-dependent synthetic lethality prediction workflow with CRISPR; MDSLP-shRNA, mutation-dependent synthetic lethality prediction workflow with shRNA; CGI, conserved synthetic lethal interactions from yeast screens.

Consistent with our expectations, different SL prediction approaches led to a diverse set of predicted SLIs. Each workflow identified more than 1,000 synthetic lethal/synthetic dosage lethal partner genes, except for CGI, which identified only 67 synthetic lethal partner genes. Predicted SLI gene sets largely did not overlap; however, functional enrichment analysis showed shared pathway involvement in the interactions identified ( Figure 4A). In particular, we found significant KEGG pathway enrichment in synthetic lethal partner genes for the cell cycle, RNA metabolism, splicing machinery, chromatin organization, and transcriptional regulatory pathways (FDR < 0.05). Interestingly, several of these results are broadly related to genomic stability maintenance, and as such, confirm previously published reports from Ku *et al.*, 2020 and others that genes involved in SLIs tend to belong to related pathways. Gene ontology biological processes (GOBPs) enriched by synthetic lethal partner genes vary, but a clustering analysis based on hierarchical structure and semantic similarity of GOBPs showed the synthetic lethal partner genes identified via the different approaches were associated with similar biological processes ( Figure 4B). A clustering analysis summarized 350 GOBPs that were initially identified via four approaches into 25 representative GOBP groups. The 25 GOBP groups are associated with synthetic lethal partner genes identified by at least two workflows, and their biological functionality is mirrored by the pathway enrichment results presented above, with enrichment in genes involved in the cell cycle, transcriptional regulation, chromatin organization, and the DNA damage response. In summary, we have demonstrated that pathway-based SL prediction is easily implemented in this framework and can quickly generate useful insights beyond the single-gene level.

### Tumor-specific SL analysis

An overlooked factor that can affect reproducibility of SLIs is context dependence. Genetic background, epigenetic cell state, and tissue type can influence synthetic lethal genetic interactions ( Moser *et al.*, 2022; Nijman & Friend, 2013; Ryan *et al.*, 2018). We show that the MDSLP workflow and our re-implementation of the DAISY algorithm can be applied to restricted subsets of the underlying data that represent samples or cell lines arising from the same cancer type. The rationale behind this type of analysis is that samples from the same cancer type could represent similar cellular origins, having a characteristic genetic interaction network that is tissue-type specific. 

**Figure 5.**
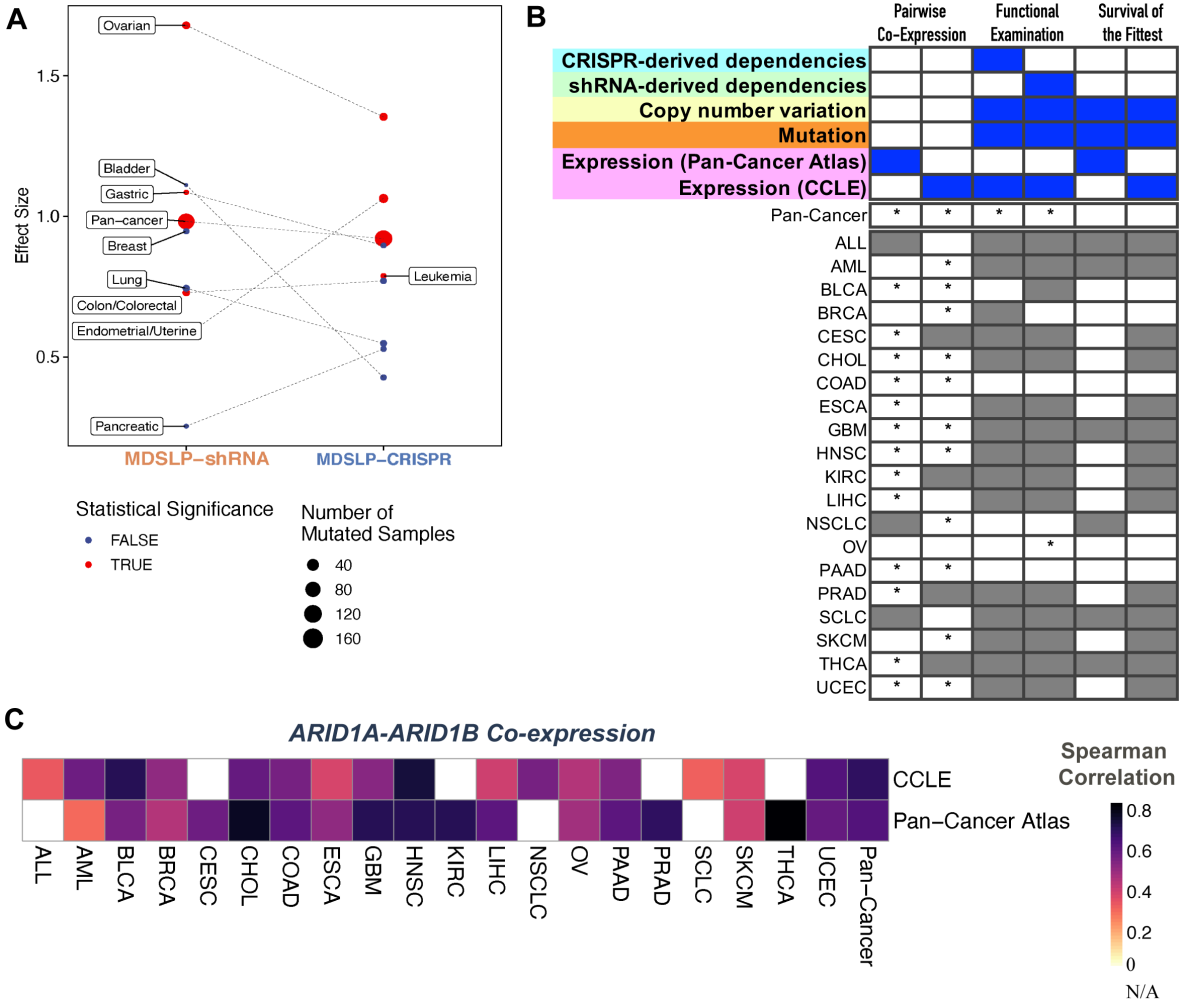
SL-Cloud enables cancer type-specific synthetic lethal inference for *ARID1A* and *ARID1B.* **A**. Evidence for synthetic lethality generated from the mutation-dependent synthetic lethality prediction (MDSLP) workflow as applied to cancer cell line dependency datasets when comparing the gene dependency scores (effects) for the shRNA and CRISPR datasets between the ARID1A-mutated group and wild-type group for different cancer types and across all cell lines (pan-cancer analysis). The threshold for statistical significance is FDR < 0.05. **B**. Statistical evidence for a synthetic lethal relationship between *ARID1A* and *ARID1B* from the DAta mIning SYnthetic lethality identification workflow (DAISY), with the results for each inference module being represented in the columns. Column heatmaps summarize the datasets used for each procedure. An asterisk (*) indicates that a test passed the FDR threshold (0.05); gray shading represents an invalid test or a lack of data availability. Gray shading represents an invalid test. **C**. Heatmap visualization depicting the Spearman correlation between *ARID1A* and *ARID1B* for the annotated cancer type for a patient derived sample (Pan-Cancer Atlas) or cancer cell line (CCLE) (rows) across different cancer types (columns).

To illustrate this principle, we investigated the previously reported SLI between *ARID1A* and *ARID1B*. Functional loss of *ARID1B* is a specific vulnerability in *ARID1A*-mutated cancers, as it affects the composition of the SWI/SNF complex ( Helming *et al.*, 2014). We applied MDLSP and DAISY to predict synthetic lethal partners for *ARID1A*. Via the MDLSP workflow, we found statistical evidence of differential dependency for *ARID1B* between *ARID1A*-mutated and wild-type cell lines in various cancer types, suggesting the potential for a SLI between the two genes across tissue types ( Figure 5A). Similar to our findings with the *BRCA*-related synthetic lethal partners, we also saw differences in the strength of the relationship based on whether *ARID1B* was knocked down via shRNA or knocked out using the CRISPR-Cas9 system. As there is strong and compelling evidence for this SLI, we find support for the interaction occurring across multiple cancer types, even if the evidence comes from shRNA-derived or CRISPR-derived dependency datasets alone.

DAISY does not predict an SLI between *ARID1A* and *ARID1B* when applied strictly, that is, when the requirement is set for statistically significant evidence across all three DAISY inference modules ( Figure 5B). However, when considering each module individually, we see strong support for the interaction, with strong positive correlation (Spearman ρ in the range [0.3 to 0.77]) between these two genes across almost all cancer types considered ( Figure 5B, C). Similar to the findings with MDSLP, we found evidence for the interaction between these two genes in ovarian cancer by using the functional examination module applied on shRNA-derived dependency dataset. This is unsurprising, as the underlying rationale for the DAISY functional examination inference procedure and MDSLP inference strategy are quite similar, and both approaches are applied to the same dataset. We found no statistical support for the interaction via genomic SoF inference, which may be explained by the fact that neither of those genes is inactivated by recurrent focal deletions that underpin that inference module.

This illustrative example showcases the flexibility of the SL-Cloud framework in that it is relatively easy to compare the results of different SL prediction approaches, while varying algorithmic parameters, using the same or different data types, or to restrict analysis to a given tumor type for further elucidation of context specificities in SL.

## Discussion

The synthetic lethality concept presents a systematic framework with which to identify and nominate potential targets for cancer treatments ( Hartwell *et al.*, 1997) Although the SL concept offers a compelling rationale to inform drug target identification, systematically testing all potential SLIs in a given tissue or disease context is experimentally intractable. SLIs are context sensitive and computational methods all have their own assumptions in search for SL pairs. Depending on the context, sample size, cohort heterogeneity and the assumptions that the computational approach relies on, researchers may get false positive and false negative results. It should be added that the very notion of false positive/negative ipso facto requires the existence of a ‘ground truth’, which is in essence contrary to the idea of context sensitivity. Our resource allows researchers to explore different SLI prioritization approaches using a wide range of multiomics and screening data. Such exploration can produce more reproducible and reliable results along with a better understanding of the predictions from different biological perspectives. Therefore, there is a continued need to develop reproducible computational inference and prioritization frameworks that make it easier to nominate the most likely SLIs for experimental follow-up or to aid in functional screen design

 In this resource we have implemented the workflows that are widely used and are the basis for other algorithms. We have reimplemented the CGI workflow because most of the computational approaches start with identifying SLIs in yeast and mapping these to their human orthologs ( Thompson *et al.*, 2015). The DAISY workflow is comprehensive, data-driven and performs SLI search from several different aspects using multiomics and screening data. It comprises individual inference modules and is extensible to handle other evidence, such as co-pathway membership, giving researchers an example of how to analyze and integrate different datasets. DAISY has been cited extensively and similar assumptions to those in DAISY have been implemented by other authors ( Das *et al.*, 2019; Liu *et al.*, 2022; Sinha *et al.*, 2017). Sinha *et al.*, 2017 claim that the DAISY algorithm is restrictive in the sense that DAISY uses a limited variety of inactivating mutations. Liu *et al.*, 2022 points out that DAISY results are non-specific pan-cancer inferences and that it doesn’t provide tissue specific results (this is an example of context sensitivity). Our resource provides a good starting point and ready to use implementations for similar research. The MDSLP workflow reflects one of the most important applications for SL discovery in cancer. Many driver mutations for different cancer types have been characterized and MDSLP can be used to identify the SL pairs for cancer driver genes, such as PARP1 - BRCA, as shown in Figure 2. The workflow can provide important clues for novel target prediction.

Here we presented a new component of ISB-CGC, SL-Cloud, that brings together computational workflows alongside large-scale datasets via cloud infrastructure to facilitate highly scalable and customizable SL analyses demonstrated through these workflows. The current implementation focuses on axes such as prevalence of genomic alterations in human samples or interactions limited to specific pathways. However, the conceptual design of the framework allows for continued modular development and extensibility. Overall, SL-Cloud offers an ensemble of methods and datasets in the cloud that enables users to collate evidence for SLIs more easily, leveraging both the richness of existing publicly available datasets and facilitating the integration of smaller user-generated custom or private datasets into the same analysis framework. From this perspective, SL-Cloud can be used for predicting SLIs for diseases other than cancer, such as viral and fungal infectious diseases ( Navare *et al.*, 2022; Usher & Haynes, 2019). The conserved genetic interaction (CGI) workflow could be used as is. The computational approaches we proposed can be used with omics and CRISPR datasets that are from fungi/virus infected samples and replacing inactive/mutant genes with genes whose activity or expression is affected by the infection. We anticipate that this resource will enable investigators to look for corroborating evidence for synthetic lethal genetic interactions with therapeutic potential and to explore such interactions in specific biological contexts.

## Data Availability

All datasets supporting the current study and relevant to SL inference are hosted on the ISB-CGC ( Reynolds *et al.*, 2017) in existing Google BigQuery tables ( Table 1). Project documentation describing how to access and use this resource are available on the project GitHub page: https://zenodo.org/badge/latestdoi/476040191.

## Software availability

Software available from: https://github.com/isb-cgc/SL-Cloud-F1000/releases/tag/F1000

Archived software as at time of publication: https://doi.org/10.5281/zenodo.6400076

License: Apache License 2.0.
