# Supplementary material for: SL-Cloud: A Cloud-based resource to support synthetic lethal interaction discovery
Source: F1000Res. 2022 Dec 22;11:493. Originally published 2022 May 4. [Version 2] doi: 10.12688/f1000research.110903.2 (PMC9880341; doi:10.12688/f1000research.110903.2)
Supplement: Peer review history

# Peer review: Reviewer response for version 2

**DOI:** 10.5256/f1000research.141975.r158530

**Published:** 2023-01-26

**Authors:** Nishanth Ulhas Nair

**Affiliations:** 1 Center for Cancer Research, National Cancer Institute, National Institutes of Health, Bethesda, USA

The authors have addressed my concerns.

Are the conclusions about the tool and its performance adequately supported by the findings presented in the article?

Yes

Is the rationale for developing the new software tool clearly explained?

Yes

Is the description of the software tool technically sound?

Yes

Are sufficient details of the code, methods and analysis (if applicable) provided to allow replication of the software development and its use by others?

Yes

Is sufficient information provided to allow interpretation of the expected output datasets and any results generated using the tool?

Yes

Reviewer Expertise:

Computational biology, cancer, genetic interactions

I confirm that I have read this submission and believe that I have an appropriate level of expertise to confirm that it is of an acceptable scientific standard.

# Peer review: Reviewer response for version 1

**DOI:** 10.5256/f1000research.122560.r151441

**Published:** 2022-10-25

**Authors:** Nishanth Ulhas Nair

**Affiliations:** 1 Center for Cancer Research, National Cancer Institute, National Institutes of Health, Bethesda, USA

In this project, the authors present SL-Cloud which provides an integrated framework of cloud-hosted data resources and curated workflows to enable facile prediction of synthetic lethal interactions. This tool may be very useful.

 I have a few minor comments for the authors to consider. 

1. It is not clear why DAISY and MDSLP were chosen for implementation when many other prediction pipelines exist. Perhaps the authors can clarify.
2. Many of the computational methods to identify synthetic lethal (SL) interactions may have false positives or false negatives. This needs to be mentioned as a limitation in the paper, so that the readers are aware of this.
3. There are some experimental double gene knockout datasets in human cancer cell lines that could be used to detect experimental SL interactions. For example: Horlbeck M, Xu A, Wang M, Bennett N, *et al. *(2018) ^1^

Are the conclusions about the tool and its performance adequately supported by the findings presented in the article?

Yes

Is the rationale for developing the new software tool clearly explained?

Yes

Is the description of the software tool technically sound?

Yes

Are sufficient details of the code, methods and analysis (if applicable) provided to allow replication of the software development and its use by others?

Yes

Is sufficient information provided to allow interpretation of the expected output datasets and any results generated using the tool?

Yes

Reviewer Expertise:

Computational biology, cancer, genetic interactions

I confirm that I have read this submission and believe that I have an appropriate level of expertise to confirm that it is of an acceptable scientific standard, however I have significant reservations, as outlined above.

## References

[1] : Mapping the Genetic Landscape of Human Cells. *Cell* .2018;174(4) : 10.1016/j.cell.2018.06.010 953-967.e22 10.1016/j.cell.2018.06.010 PMC642645530033366

# Response

**Published:** 2022-12-12

**Authors:** Nyasha Chambwe

**Comment:** *It is not clear why DAISY and MDSLP were chosen for implementation when many other prediction pipelines exist. Perhaps the authors can clarify*

 The following paragraph has been added to the Discussion section:

 “In this resource we have implemented the workflows that are widely used and are the basis for other algorithms. We have reimplemented the CGI workflow because most of the computational approaches start with identifying SLIs in yeast and mapping these to their human orthologs (Thompson *et al.*, 2015). The DAISY workflow is comprehensive, data-driven and performs SLI search from several different aspects using multiomics and screening data. It comprises individual inference modules and is extensible to handle other evidence, such as co-pathway membership, giving researchers an example of how to analyze and integrate different datasets. DAISY has been cited extensively and similar assumptions to those in DAISY have been implemented by other authors (Das *et al.*, 2019; Liu *et al.*, 2022; Sinha *et al.*, 2017). Sinha *et al.*, 2017 claim that the DAISY algorithm is restrictive in the sense that DAISY uses a limited variety of inactivating mutations. Liu *et al.*, 2022 points out that DAISY results are non-specific pan-cancer inferences and that it doesn’t provide tissue specific results (this is an example of context sensitivity). Our resource provides a good starting point and ready to use implementations for similar research. The MDSLP workflow reflects one of the most important applications for SL discovery in cancer. Many driver mutations for different cancer types have been characterized and the MDSLP can be used to identify the SL pairs for cancer driver genes, such as PARP1 - BRCA, as shown in Fig 2.  The workflow can provide important clues for novel target prediction.” 

**Comment:** *Many of the computational methods to identify synthetic lethal (SL) interactions may have false positives or false negatives. This needs to be mentioned as a limitation in the paper, so that the readers are aware of this.*

 The following sentences have been added to the Discussion section:

 “SLIs are context sensitive and the computational methods all have their own assumptions in search for SL pairs. Depending on the context, sample size, cohort heterogeneity and the assumptions that the computational approach relies on, researchers may get false positive and false negative results. It should be added that the very notion of false positive/negative ipso facto requires the existence of a ‘ground truth’, which is in essence contrary to the idea of context sensitivity. Our resource allows researchers to explore different SLI prioritization approaches using a wide range of multiomics and screening data. Such exploration can produce more reproducible and reliable results along with a better understanding of the predictions from different biological perspectives.”

**Comment:** *There are some experimental double gene knockout datasets in human cancer cell lines that could be used to detect experimental SL interactions. For example: Horlbeck M, Xu A, Wang M, Bennett N, et al. (2018)*

 We have cited the paper in the proper context, where we mention CRISPR perturbations for SLI identification:

 “A recent study  detects human SLIs based on systematic CRISPRi perturbation of over two thousand gene pairs” sentence has been added in the second paragraph of the Introduction and Horlbeck M, Xu A, Wang M, Bennett N, *et al. *(2018)

# Peer review: Reviewer response for version 1

**DOI:** 10.5256/f1000research.122560.r144718

**Published:** 2022-08-18

**Authors:** Jane Usher

**Affiliations:** 1 University of Exeter, Exeter, UK

In this paper by Tercan *et al*., the authors present a synthetic-lethal cloud-based component of the ISB-CGC that can provide an upgraded and integrated framework of cloud-based data resources and workflows to enable and enhance the prediction of synthetic lethal interactions in cancer tissue networks. This tool is devised in order to address the challenge of pre-processing large datasets from multiple sources and the availability to perform multiple prediction comparisons. The SL-cloud allows users to customise inputs and then test predictions of SLIs approaches across multiple datasets and add in new studies. This tool currently is focused on use by cancer biology researchers in support of the development of potential drug targets, such as the case for BRAC1/2 and PARP1.

 It would interesting if the authors could expand more in the discussion or provide an example that is not already shown in previous publications of how this tool may be modified to other organisms, such as fungal pathogens where a slue of SLI studies are undertaken with many times laborious workflows.

 Overall, this was an exciting paper to read and very written with a timely concept.

Are the conclusions about the tool and its performance adequately supported by the findings presented in the article?

Yes

Is the rationale for developing the new software tool clearly explained?

Yes

Is the description of the software tool technically sound?

Yes

Are sufficient details of the code, methods and analysis (if applicable) provided to allow replication of the software development and its use by others?

Yes

Is sufficient information provided to allow interpretation of the expected output datasets and any results generated using the tool?

Yes

Reviewer Expertise:

Fungal biology; molecular biology, microbiology, chemogenomic and gentic interaction screens.

I confirm that I have read this submission and believe that I have an appropriate level of expertise to confirm that it is of an acceptable scientific standard.

# Response

**Published:** 2022-12-12

**Authors:** Nyasha Chambwe

We added the following text to the Discussion section:

 “From this perspective, SL-Cloud  can be used for predicting SLIs for diseases other than cancer, such as viral and fungal infectious diseases. The conserved genetic interaction (CGI) workflow could be used as is. The computational approaches we proposed can be used with omics and CRISPR datasets that are from fungi/virus infected samples and replacing inactive/mutant genes with genes whose activity or expression is affected by the infection.”
